# Progranulin Protects Vascular Endothelium against Atherosclerotic Inflammatory Reaction via Akt/eNOS and Nuclear Factor-κB Pathways

**DOI:** 10.1371/journal.pone.0076679

**Published:** 2013-09-30

**Authors:** Hwan-Jin Hwang, Tae Woo Jung, Ho Cheol Hong, Hae Yoon Choi, Ji-A Seo, Sin Gon Kim, Nan Hee Kim, Kyung Mook Choi, Dong Seop Choi, Sei Hyun Baik, Hye Jin Yoo

**Affiliations:** Division of Endocrinology and Metabolism, Department of Internal Medicine, College of Medicine, Korea University, Seoul, Korea; UAE University, Faculty of Medicine & Health Sciences, United Arab Emirates

## Abstract

**Objective:**

Atherosclerosis is considered a chronic inflammatory disease, initiated by activation and dysfunction of the endothelium. Recently, progranulin has been regarded as an important modulator of inflammatory processes; however, the role for prgranulin in regulating inflammation in vascular endothelial cells has not been described.

**Method and Results:**

Signaling pathways mediated by progranulin were analyzed in human umbilical vein endothelial cells (HUVECs) treated with progranulin. Progranulin significantly induced Akt and endothelial nitric oxide synthase (eNOS) phosphorylation in HUVECs, an effect that was blocked with Akt inhibitor. Furthermore, nitric oxide (NO) level, the end product of Akt/eNOS pathway, was significantly upregulated after progranulin treatment. Next, we showed that progranulin efficiently inhibited lipopolysaccharide (LPS)-mediated pro-inflammatory signaling. LPS-induced phosphorylation of IκB and nuclear factor-κB (NF-κB) levels decreased after progranulin treatment. Also, progranulin blocked translocation of NF-κB from the cytosol to the nucleus. In addition, progranulin significantly reduced the expression of vascular cell adhesion molecule-1 (VCAM-1) and intercellular adhesion molecule-1 (ICAM-1) by inhibiting binding of NF- κB to their promoter regions and blocked attachment of monocytes to HUVECs. Progranulin also significantly reduced the expression of tumor necrosis factor receptor-α (TNF-α) and monocyte chemo-attractant protein-1 (MCP-1), the crucial inflammatory molecules known to aggravate atherosclerosis.

**Conclusion:**

Progranulin efficiently inhibited LPS-mediated pro-inflammatory signaling in endothelial cells through activation of the Akt/eNOS pathway and attenuation of the NF-κB pathway, suggesting its protective roles in vascular endothelium against inflammatory reaction underlying atherosclerosis.

## Introduction

Inflammation is the pivotal pathologic mechanism of atherosclerosis and contributes to all of its stages, from plaque initiation to growth and rupture [1]. The inflammatory state within atherosclerotic plaques is a stronger predictor of acute cardiovascular events than the degree of structural stenosis [2]. Recently, progranulin has received attention as an important modulator of the inflammatory process. Progranulin, also known as proepithelin or acrogranin, is a secreted glycoprotein that consists of 7.5 granulin domains [3]. It is widely expressed in mammalian tissues and plays a critical role in multiple physiological and pathological conditions, including cell growth, wound healing, and tumorigenesis [4]. Tang et al. [5] demonstrated that progranulin directly binds to tumor necrosis factor receptor (TNFR) and disturbs TNF-α-activated intracellular signaling; treatment of bone marrow-derived macrophages with recombinant progranulin inhibits TNF-α-induced phosphorylation of p38 and c-jun N-terminal kinase (JNK) and impairs nuclear factor-κB (NF-κB) nuclear translocation. Previously, Zhu et al. [6] reported that full-length progranulin suppresses TNF-α-induced neutrophil activation, and progranulin-deficient macrophages challenged with lipopolysaccharide (LPS) increase pro-inflammatory cytokine production [7], suggesting its beneficial anti-inflammatory effects.

Progranulin has a high probability of being involved in subclinical inflammatory disorders associated with human obesity and type 2diabetes. However, there have been few studies examining the implication of progranulin in obesity-related metabolic disorders. Youn et al. [8] found, for the first time, that progranulin serum concentration was significantly higher in type 2 diabetic subjects compared to those with normal glucose tolerance, and the difference in progranulin levels correlated with macrophage infiltration in omental adipose tissue. They suggested that progranulin contributes to cross-talk between adipocytes and macrophages in adipose tissue, promotes adipocyte inflammatory responses, and recruits monocytes into adipose tissue [8]. Very recently, Matsubara et al. [9] redefined progranulin as a key adipokine mediating high fat diet-induced insulin resistance and obesity in adipose tissue. They demonstrated that progranulin suppresses insulin-stimulated glucose uptake in 3T3-L1 adipocytes, and that ablation of progranulin in mice prevents high-fat diet-induced elevation of interlukin-6 in blood and adipose tissue [9], implying its pro-inflammatory role in metabolic disorders. Taken together, the previous studies suggest that progranulin may play dual roles in the inflammatory process by exerting either anti-inflammatory or pro-inflammatory functions depending on the target tissue. However, to our knowledge, there has been no research examining the function of progranulin on the atherosclerotic process, a representative inflammatory disorder, in endothelial cells.

Therefore, we investigated a possible effect of progranulin on the inflammatory process underlying atherosclerosis in human umbilical vein endothelial cells (HUVECs). We asked if progranulin exerts its effect through regulation of 1) the Akt-endothelial nitric oxide synthase (eNOS) signaling pathway; 2) NF-κB-dependent expression of vascular cell adhesion molecule-1 (VCAM-1), intercellular adhesion molecule-1 (ICAM-1), monocyte chemo-attractant protein-1 (MCP-1), and TNF-α; and 3) adhesion of monocytes to endothelial cells.

## Methods

### Materials

Sources of materials were as follows: anti-VCAM-1 (1:500 dilution), anti-ICAM-1 (1:500 dilution), anti-Lamin B1 (1:500 dilution), anti-beta actin (1:5,000 dilution; Santa Cruz Biotechnology, Santa cruz, CA); anti-pAkt (1:1,000 dilution), anti-total Akt (1:2,000 dilution), anti-pIκB (1:1,000 dilution), anti-total IκB (1:2,000 dilution), anti-pNF-κB (1:1,000 dilution), anti-total NF-κB (1:2,000 dilution), anti-p-eNOS (1:1,000 dilution), and anti-total eNOS (1:2,000 dilution; Cell Signaling Technology, Boston, MA); lipopolysaccharide (LPS) from *Escherichia* *coli*055:B5, anti-mouse IgG-FITC, Propidium iodide (PI) dye, Akt kinase inhibitor, and gelatin solution (Sigma Aldrich, St. Louis, MO, U.S.); progranulin (Adipogen International, South Korea); DokDo-MARK^TM^ pre-stained protein marker (Elpis Biotech, Seo-gu, Daejun, South Korea); and Triton X-100 (Biosesang, Sungnam, South Korea).

### Cell Culture

HUVECs from Invitrogen Life Technologies (Carlsbad, CA, U.S.), listed as C-003-5C, were cultured on 0.2% gelatin-coated dishes with M200PRF media (Invitrogen, U.S.) containing Low Serum Growth Supplement (Invitrogen, U.S.) and 10% fetal bovine serum (FBS; Invitrogen, U.S.). The cells were used at passage 3 to 6 for experiments. THP-1 cells from Korean cell line bank (KCLB, Seoul, South Korea) were maintained in RPMI 1640 (Invitrogen, U.S.) containing 10% FBS, 50 U/mL penicillin and 50 µg/mL streptomycin (Invitrogen, U.S.), and used at passages 5 to 6 for experiments.

### Western Blot Analysis

Cell extracts were separated by SDS-PAGE and transferred to 0.45 µm nitrocellulose membranes (Amersham Bioscience, Westborough, MA, U.S.). The membranes were blocked in 0.05% TBST containing 5% non-fat dry milk or 5% BSA for 1 hour at room temperature. The blocked membranes were incubated with primary antibody (1:500–2000) followed by horseradish peroxidase-conjugated secondary antibody (1:5000; Amersham Bioscience, U.S.). The membranes were developed using chemiluminescence solution (Bio-Rad Laboratories, CA, U.S.).

### Measurement of NO Level

HUVECs were starved in M200PRF media containing 1% FBS for 3 hour. The starved cells were incubated with 200 ng/mL of PGRN, and NO levels were analyzed from the culture media after 1 or 6 hour. NO production was measured using a Nitric Oxide Colorimetric Assay Kit (BioVision, Mountain View, CA, U.S.), and OD values were measured at 540 nm.

### Quantitative Real-time PCR (qPCR)

HUVECs were stimulated with LPS (200 ng/mL) or LPS and PGRN (200 ng/mL) for 1 hour. Total RNA was separated using Trizol (Invitrogen, U.S.) and used for cDNA synthesis. Primer sets for qPCR were as follows: TNF-α, 5’-TGC TGC ACT TTG GAG TGA TCG-3’ (forward) and 5’-TGT CAC TCG GGG TTC GAG AAG-3’ (reverse); MCP-1, 5’-GAT GCA ATC AAT GCC CCA GTC-3’ (forward) and 5’-TCC TTG GCC ACA ATG GTC TTG-3’ (reverse); and β-actin, 5’-CGC AAA GAC CTG TAC GCC AAC-3’ (forward) and 5’-CAC GGA GTA CTT GCG CTC AGG-3’ (reverse). Thermal profile conditions were as follows: pre-incubation at 95°C for 10 min followed by 40 cycles of amplification at 95°C for 10s and 60°C for 20 s. TNF-α and MCP-1 transcript levels were calculated by the 2-ΔΔCT method.

### Chromatin Immunoprecipitation (ChIP) Assay

Cells were incubated with LPS (200 ng/mL) or LPS and progranulin (200 ng/mL) for 15 minutes and then fixed with 1% formaldehyde. The ChIP assay was performed on the fixed cells using an EZ-ChIP^TM^ Chromatin Immunoprecipitation Kit (Millipore Corporation, Billerica, MA, U.S.) according to the instruction manual. Primer sets for PCR were as follows: ICAM-1 [10], 5’-ACC TTA GCG CGG TGT AGA CC-3’ (forward) and 5’- CTC CGG AAC AAA TGC TGC-3’ (reverse); VCAM [11], 5’- AAA TCA ATT CAC ATG GCA TA-3’ (forward) and 5’-AAG GGT CTT GTT GCA GAG G-3’ (reverse).

### Immunofluorescence Microscopy Analysis

Cells were seeded in 96-well plates and cultured for 24 hour, incubated with LPS (200 ng/mL) or LPS and progranulin (200 ng/mL) for 15 minutes, and then fixed with 3.8% formaldehyde for 10 minutes. After washing with PBS, the fixed cells were permeabilized with pre-chilled MeOH for 10 minutes at -20°C and then incubated in blocking buffer (PBS containing 1% BSA and 0.2% triton X-100) for 1 hour. The cells were incubated in dilution buffer (PBS containing 1% BSA and 0.05% triton X-100) containing NF-κB antibody for 16 hour at -20°C, washed, and then incubated in dilution buffer containing FITC-conjugated anti-mouse lgG for 1 hour at room temperature. The FITC-labeled cells were washed, and propidium iodide was used to stain the nuclei.

### Protein Fractionation

HUVECs were stimulated with LPS (200 ng/mL) or LPS and progranulin (200 ng/mL) for 15 minutes, and then fractionated by using protein fractionation kit (Biovision, Mountain View, CA) according to manufacturer’s direction.

### Monocyte Adhesion Assay

HUVECs were incubated in 12-well plates for 24 hour and subsequently stimulated with LPS (200 ng/mL) or LPS and progranulin (200 ng/mL) for 6 h. THP-1 cells were labeled with 10 µg/mL of the fluorescent dye BCECF/AM (2’, 7’-bis(2-carboxyethyl)-5(6)-carboxyfluorescein acetoxymethyl ester, Invitrogen, U.S.) at 37°Cfor 30 minutes. The fluorescent-labeled THP-1 cells were washed 3–5 times with PBS containing 2% FBS and resuspended in RPMI 1640 medium. Then, the pre-labeled THP-1 cells (2 × 10^6^/well) were co-cultured with stimulated HUVECs at 37°C for 30 minutes. In one group, the cells were washed 3–5 times with PBS containing 2% FBS and analyzed under a fluorescence microscope. In other 4 groups, the cells were washed and incubated in PBS with 0.5% Triton X-100. Cell lysates were analyzed for total fluorescence level using a spectrofluorometer (PerkinElmer, Bridgeville, PA, U.S.) at 485nm/520nm.

### Statistical Analysis

All analyses were performed using Sigma Plot 2001 for Windows, and results are expressed as mean ±SD. Student’s *t*-test was used for statistical analyses. Data were obtained for a minimum of three experiments and considered significant at *P*< 0.05.

## Results

### Progranulin Up-regulated Akt/eNOS Phosphorylation Level in HUVECs

**Figure 1:**
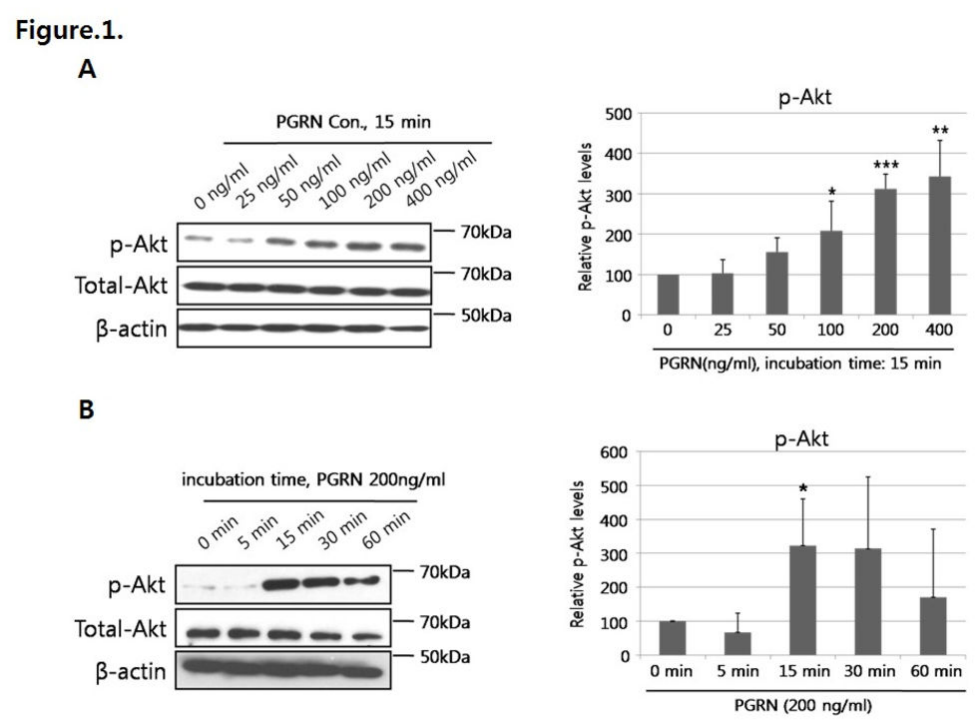
Progranulin (PGRN) induced Akt phosphorylation in HUVECs. (A) Cells were stimulated with various doses of progrnaulin (0, 25, 50, 100, 200, or 400 ng/mL) for 15 min and analyzed by Western blotting for phosphorylated-Akt (p-Akt) level. (B) HUVECs were incubated with 200 ng/mL of progranulin for the indicated times (0, 5, 15, 30, or 60 min). Data were obtained from three separate experiments. Error bars represent mean ± SD (*, *P*< 0.05, *t*-test).

**Figure 2:**
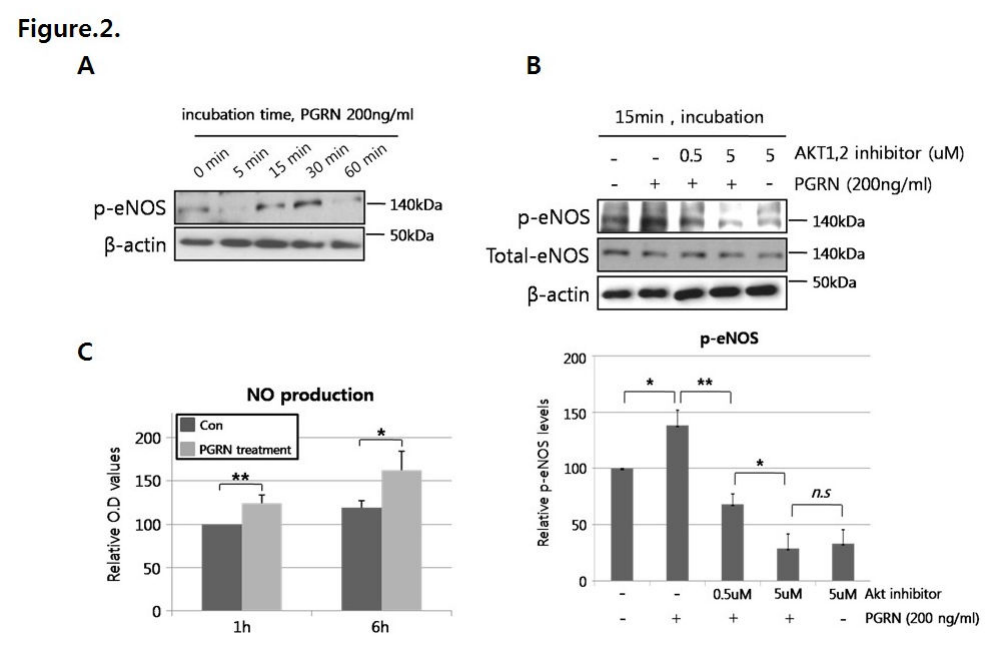
Progranulin (PGRN) induced activation of the Akt/eNOS/NO pathway in HUVECs. (A) Cells were incubated with 200 ng/mL of progranulin for 0, 5, 15, 30, or 60 min and analyzed by Western blotting for phosphorylated-eNOS (p-eNOS) level. (B) HUVECs were stimulated with 200 ng/mL of progranulin with or without Akt inhibitor (0.5 or 5 µM) for 15 min, and p-eNOS level was measured by Western blotting. (C) Cells were incubated with 200 ng/mL of progranulin for the indicated times. NO production was analyzed from culture media using the Griess reagent, and OD values were measured at 540nm. Data were obtained from three separate experiments. Error bars represent mean ±SD (n.s.: not significant; *, *P*< 0.05, **, *P*< 0.005, *t*-test)..

Western blot analysis showed that Akt phosphorylation level was markedly induced by progranulin treatment in a dose dependent manner (Figure 1A). When HUVECs were stimulated with 200 ng/mL of progranulin for 0, 5, 15, 30 or 60 min, Akt phosphorylation levels peaked after 15–30 min of treatment and decreased after 60 min (Figure 1B). eNOS, one of the downstream targets of Akt, is phosphorylated after Akt activation. To confirm that eNOS was activated in response to progranulin-mediated Akt phosphorylation, we identified phosphorylated eNOS (p-eNOS) levels in progranulin-treated HUVECs by Western blotting. The level of p-eNOS was significantly increased after 15–30 min of progranulin treatment (Figure 2A), and this change disappeared after treatment with Akt inhibitor (Figure 2B). Through the Akt/eNOS pathway, nitric oxide (NO) was significantly increased after treatment with progranulin (Figure 2C).

### Progranulin Inhibited NF-κB phosphorylation Induced by LPS

**Figure 3:**
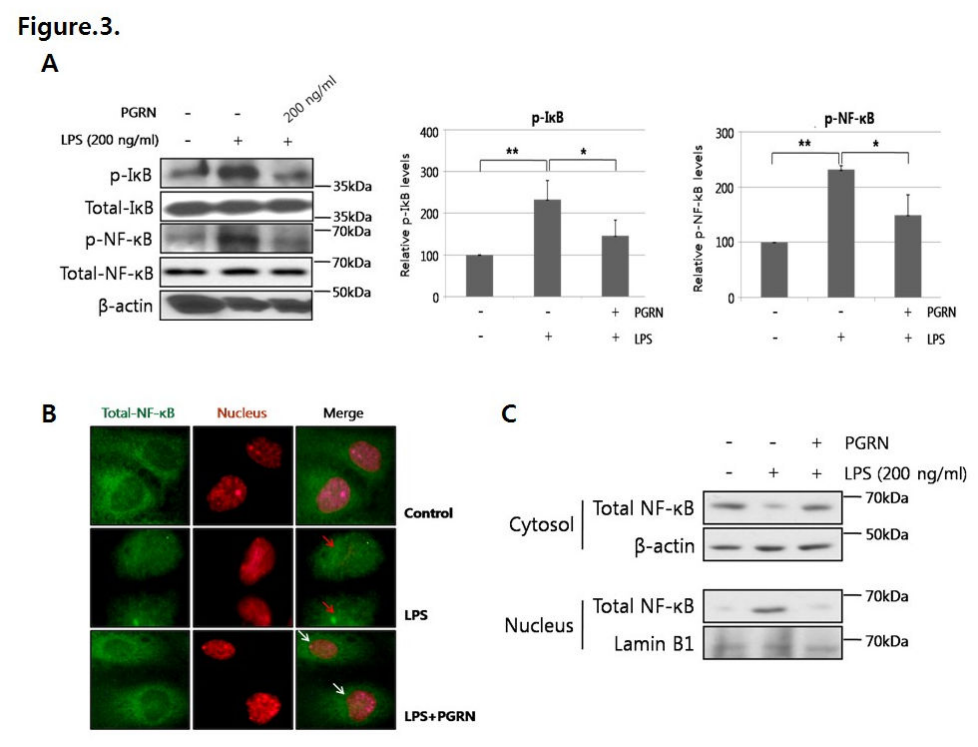
Progranulin(PGRN) inhibited LPS-mediated IκB and NF-κB activation. (A) Cells were incubated with LPS (200 ng/mL) or LPS and PGRN (200 ng/mL) for 15 min and analyzed by Western blotting for phosphorylated-IκB (p-IκB) and -NF-κB (p-NF-κB) levels. Data were obtained from three separate experiments. Error bars represent mean ±SD (*, *P*< 0.05, **, *P*< 0.005, *t*-test). (B) Immunofluorescence microscopy analysis was performed for identification of NF-κB location after 15 min of LPS or LPS and progranulin treatment. NF-κB was labeled by green fluorescence (FITC), and nucleus was stained by red fluorescence (propidium iodide). The *red* *arrows* indicate that NF-κB was translocated from the cytosol to the nucleus by LPS, and the *white* *arrows* mean that progranulin inhibited NF-κB translocation from the cytosol to the nucleus. (C) Cytosol and nuclear protein of LPS or LPS and PGRN-treated HUVEC were separated, and analyzed by western blotting for NF-κB translocation.

Phosphorylation levels of IκB and NF-κB were significantly increased in HUVECs treated with LPS. However, the levels were significantly decreased in HUVECs co-treated with LPS and progranulin (Figure 3A). Through fluorescence microscopy analysis, we demonstrated translocation of NF-κB from the cytosol to the nucleus after LPS treatment (Figure 3B; red arrows), whereas NF-κB translocation was efficiently inhibited in HUVECs co-treated with LPS and progranulin (Figure 3B; white arrows). In addition, we rechecked NF-κB location by using nuclear and cytosolic protein fractionation kit, suggesting that progranulin significantly decreased the nucleus-located NF-κB expression by LPS (Figure 3C).

### Progranulin Decreased Expression of Adhesion Molecules and Adhesion of Monocytic Cells to HUVECs Through an NF-κB-dependent Pathway

**Figure 4:**
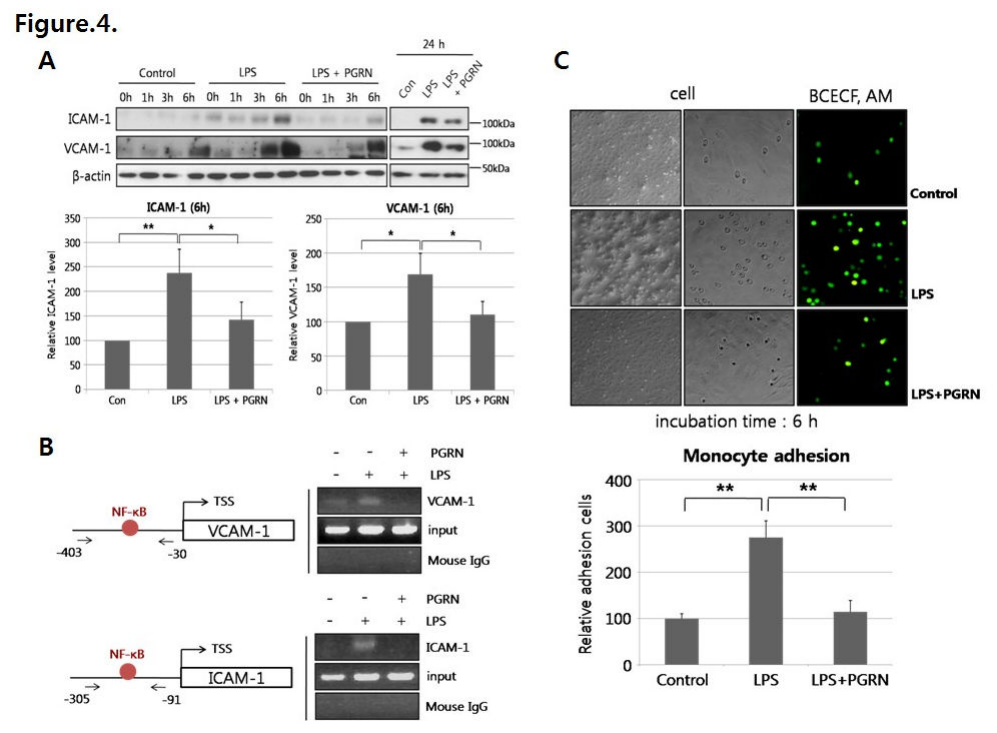
Progranulin (PGRN) blocked monocyte adhesion to HUVECs through down-regulation of adhesion molecules. (A) Cells were stimulated with LPS (200 ng/mL) or LPS and progranulin (200 ng/mL) for the indicated times (0, 1, 3, 6, or 24 h). VCAM-1 and ICAM-1 expression levels were measured by Western blotting. (B) NF-κB binding to VCAM-1 and ICAM-1 promoter regions was identified by the ChIP assay. *TSS* means transcription start site. (C) HUVECs were incubated with LPS or LPS and progranulin for 6 h and subsequently co-cultured with fluorescent (BCECF/AM)-labeled THP-1 for 30 min. The adhesion ability of THP-1 cells was measured using a fluorescence microscope and spectrofluorometer. Error bars represent mean ±SD (*, *P*< 0.05, **, *P*< 0.005, *t*-test).

**Figure 5:**
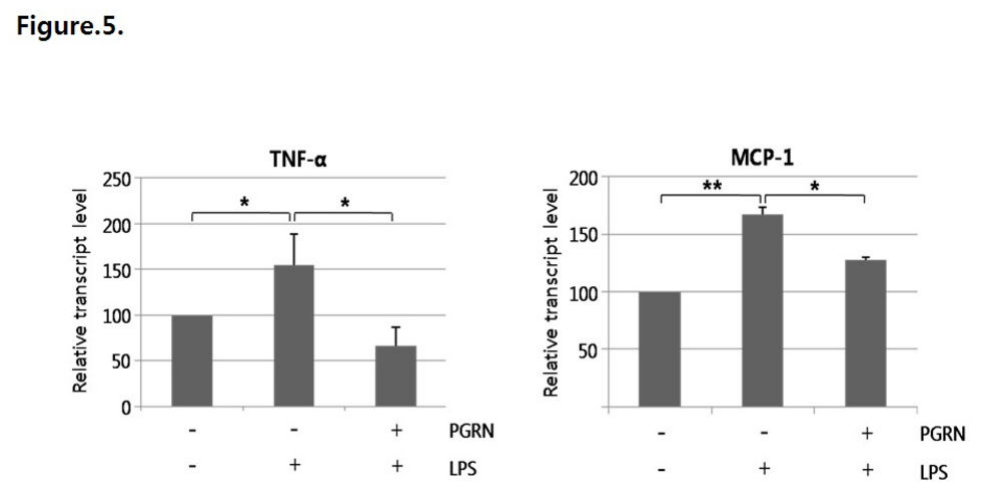
Progranulin (PGRN) blocked LPS-inducedTNF-α and MCP-1 transcription. Progranulin efficiently reduced transcript levels of TNF-α and MCP-1. qPCR was performed for analysis of TNF-α and MCP-1 expression levels. Data were obtained from three separate experiments. Error bars represent mean ±SD (*, *P* < 0.05, **, *P* < 0.005, *t*-test).

VCAM-1 and ICAM-1 expression levels were significantly increased after 6 h of LPS treatment in our experiments. We showed that progranulin significantly decreased LPS-induced VCAM-1 and ICAM-1 expression (Figure 4A) by inhibiting binding of NF-κB to the promoter regions of VCAM-1 and ICAM-1 (Figure 4B). In addition, VCAM-1 and ICAM-1 were associated with the adhesion ability of monocytes. THP-1 cells, a monocyte cell line, showed increased adhesion ability to HUVECs treated with LPS compared with controls, whereas adhesion of THP-1 cells to HUVECs was markedly blocked by co-treatment with LPS and progranulin (Figure 4C). Lastly, progranulin significantly reduced LPS-mediated expression of TNF-α and MCP-1, the crucial inflammatory molecules known to aggravate the atherosclerotic inflammatory process (Figure 5).

## Discussion

Progranulin is known as an important modulator in various inflammatory processes such as neurodegenerative dementia and rheumatoid arthritis [5,12]. Recently, it has been regarded as a novel adipokine to provoke insulin resistance [9], but its function in vasculature is nearly unknown. In the present study, we examined for the first time the effects of progranulin on the inflammatory atherosclerotic process in human vascular endothelial cells. The major finding of our study is that progranulin exerts anti-inflammatory functions in vascular endothelial cells by activating the Akt/eNOS signaling pathway and suppressing LPS-induced expression of VCAM-1, ICAM-1, MCP-1, and TNF-α and monocyte migration via inhibition of the NF-κBpathway.

Because progranulin has been shown to be involved in the PI3K/Akt signaling pathway [13,14], we preferentially examined the effect of progranulin on the Akt/eNOS signaling pathway in human endothelial cells. Endothelial dysfunction, characterized by decreased production of NO, is an early and key mediator that links obesity and cardiovascular diseases [15]. Nitric oxide, a vasodilator synthesized by eNOS, protects the vasculature by inhibiting platelet aggregation, monocyte adhesion, and smooth muscle cell proliferation [16]. In the present study, progranulin increased eNOS phosphorylation and NO level, and this effect disappeared after treatment with Akt inhibitor, suggesting an anti-atherogenic effect of progranulin via the Akt/eNOS pathway.

Furthermore, we showed that progranulin inhibited LPS-induced phosphorylation of NF-κB and translocation of NF-κB to the nucleus. NF-κB, for a long time, has been known to play a crucial role in the regulation of inflammatory reactions in atherosclerosis. Activated NF-κB has been found in macrophages, smooth muscle cells, and endothelial cells in human atherosclerotic lesions but not in healthy vessels [17]. NF-κB activation by various inflammatory cytokines results in the induction of endothelial adhesion molecules including VCAM-1, ICAM-1, and E-selectin, which play an important role in recruiting leukocytes to activated endothelium [18]. In the present study, progranulin decreased the expression of VCAM-1, ICAM-1, and MCP-1 and migration of monocytes to endothelial cells by inhibiting NF-κB from binding to the promoter regions of these molecules. Recently, Tang et al. [5] showed that progranulin prevents inflammation in multiple arthritis mouse models and impairs gene expression activated by NF-κB. An engineered protein made of three progranulin fragments, atsttrin, exerts it protective effects in the pathogenesis of inflammatory arthritis by acting as an antagonist of TNF-α-mediated inflammatory responses [19]. Furthermore, Yin et al. [7] found that neurons from progranulin-deficient mice were more vulnerable to damage by neuroinflammation, implying its role in neuronal integrity. Mutations in progranulin were discovered to be a cause of frontotemporal lobar degeneration, the second most common presenile dementia disorder after Alzheimer’s disease [20]. When compared with neurodegenerative diseases and arthritis; there have been a limited number of studies examining the function of progranulin in vascular diseases. Until now, only Kojima et al. [21] have identified the expression of progranulin in vascular smooth muscle cells (VSMCs) and macrophages in plaques using immunohistochemical analysis of human carotid endoatherectomy specimens. In that study, exogenous progranulin suppressed interleukin-8 (IL-8) production from VSMCs, but knockdown of progranulin increased TNF-α-induced IL-8 secretion, an important cytokine involved in the progression of atherosclerosis. Similarly, we showed that progranulin is beneficial in preventing the inflammatory process in endothelial cells.

However, not all the actions of progranulin have been reported as anti-inflammatory. Youn et al. [8] showed that elevated progranulin serum concentration is associated with visceral obesity, macrophage infiltration in omental adipose tissue, and dyslipidemia, suggesting its role as a novel marker of chronic inflammation in obesity. Moreover, a recent study identified progranulin as a key adipokine that triggers systemic insulin resistance, adipocyte hypertrophy, and obesity through production of IL-6 in adipose tissue [9]. Previously, we also reported that circulating progranulin concentration has a significant positive correlation with systemic inflammatory markers such as hsCRP and IL-6 [22] and Yilmaz et al. [23] showed that progranulin is significantly higher in nonalcoholic fatty liver disease patients than in controls, and progranulin expression correlates with the degree of hepatic fibrosis.

Therefore, the physiological function of progranulin seems to be very complicated, exerting pro-inflammatory or anti-inflammatory functions in different tissues. After secretion, progranulin is cleaved into smaller peptides called granulins. Full-length progranulin is generally anti-inflammatory, whereas proteolytically-cleaved granulin has the opposite effect. Granulin peptides increase the expression of pro-inflammatory cytokines, while progranulin is a potent inhibitor of inflammatory cytokines and promotes up-regulation of Th2 cytokines such as IL-4 and IL-10 [24,25]. During the inflammatory process, immune cells release proteases that can digest progranulin into individual granulin domains, which are pro-inflammatory and neutralize the anti-inflammatory effect of intact progranulin [24,26]. Thus, factors controlling the conversion of full-length progranulin into granulin peptides appear to be pivotal. Okura et al. [27] showed that high density lipoprotein binds to progranulin and suppresses its conversion into pro-inflammatory granulins. Although the present study was unable to not address the underlying mechanism of progranulin degradation into granulin peptides in endothelial cells, it will be an important area of research for understanding the pathophysiology of atherosclerosis.

**Figure 6:**
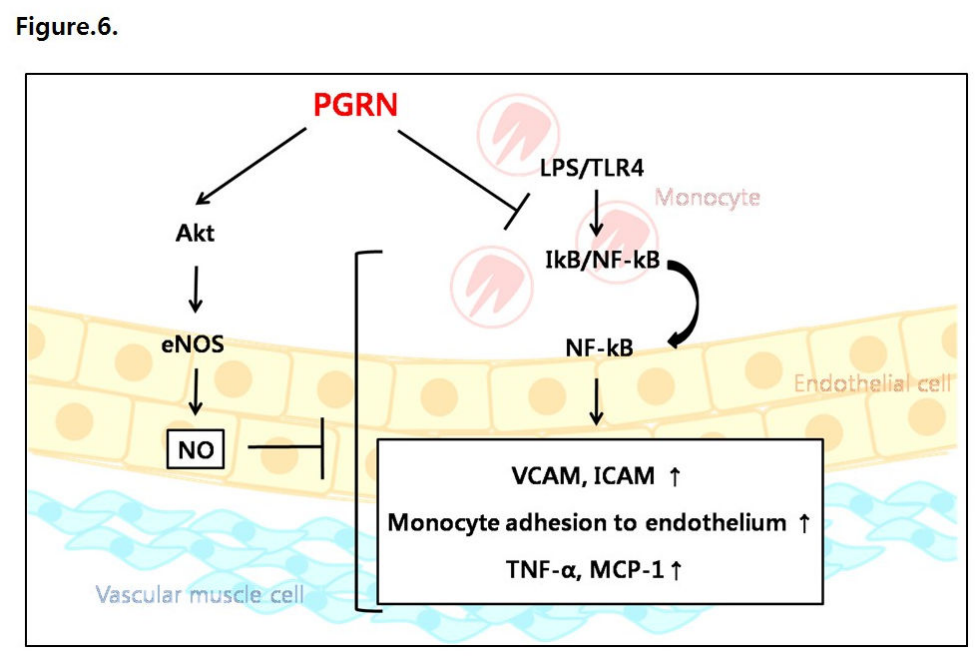
Schematic diagram of the progranulin (PGRN) mechanism in HUVECs. Progranulin activates (◊) the Akt/eNOS/NO pathway and inhibits () LPS/TLR4-mediated pro-inflammatory effects.

In conclusion, progranulin effectively inhibited the atherosclerotic inflammatory process induced by LPS in human endothelial cells via activation of the Akt/eNOS pathway and attenuation of the NF-κB pathway (Figure 6). These results suggest that progranuln might be utilized as a novel therapeutic target for atherosclerosis. Further *in vivo* and clinical studies are warranted to reinforce our current findings.
